# Lemierre syndrome: case report

**DOI:** 10.1590/1677-5449.002418

**Published:** 2018

**Authors:** Rodrigo de Oliveira Veras, Linda Luísa Barasuol, Carolina Pedrassani de Lira, Flávia Caroline Klostermann, Lourenço Sabo Müller, Luiz Eduardo Nercolini, Gustavo Fabiano Nogueira

**Affiliations:** 1 Faculdade Evangélica do Paraná – FEPAR, Curitiba, PR, Brasil.; 2 Hospital Universitário Evangélico de Curitiba – HUEC, Curitiba, PR, Brasil.; 3 Instituto Neurológico de Curitiba – INC, Curitiba, PR, Brasil.

**Keywords:** Lemierre syndrome, tooth extraction, thrombophlebitis, pulmonary embolism

## Abstract

Lemierre syndrome is characterized by septic thrombophlebitis of the internal jugular vein, after an oropharyngeal infection, with septic embolization to the lungs or other organs. This case report describes a 37-year-old female patient who presented with edema and pain in the right hemiface with onset 3 days previously and progressive fatigue and dyspnea since the previous day. She had had tooth 48 extracted 3 days previously. Physical examination at admission found tachypnea, with 60% saturation (in room air), edema at the angle of the right mandible, diffuse reduction of vesicular murmur, and calves free from clubbing. Angiotomography of the chest and laboratory tests were compatible with septic emboli, and cervical computed tomography confirmed a diagnosis of septic thrombophlebitis of the internal jugular vein. She was managed with antibiotics and given treatment for her symptoms. Lemierre syndrome most often occurs in young men and there is embolization to the lungs in up to 97% of cases. Rarely, the etiology of this syndrome may be tooth extraction. Computed tomography is the imaging method most often used for diagnosis and treatment is basically antibiotic. Surgery is thus rarely necessary.

## INTRODUCTION

 Lemierre syndrome (LS) is characterized by septic thrombophlebitis of the internal jugular vein after oropharyngitis, with septic embolization to the lungs or other organs. 1 ^-^ 3 In extremely rare cases, tooth extraction may also trigger this syndrome. 4 ^,^ 5

 The syndrome primarily affects young adults 6 and can potentially be fatal. 1 ^,^ 2 ^,^ 4 ^,^ 7 Lemierre syndrome is also known as the “forgotten disease”, because of its rarity. 7 It has an incidence of around 3.6 million people per year, 2 with mortality of around 5%, when diagnosed. 5 ^,^ 8 Treatment is basically founded on antibiotic therapy tailored to the pathogen involved and surgery is rarely needed. 6

 In this case report, we describe a woman who exhibited LS atypically, after tooth extraction. 

## CASE REPORT

 A 37-year-old female patient was admitted after presenting at a hospital on April 1, 2017 with facial edema and pain involving the right hemiface, with onset 3 days previously and asthenia and progressive dyspnea in response to moderate force since the previous day. She reported no episodes of fever. Hitherto healthy, she had performed her daily physical activities with no complaints prior to this occurrence. She had a history of bruxism, complicated by a dental trauma to the right lower second molar 3 days previously, requiring extraction, which had been performed immediately. 

 Her general state of health was normal on physical examination, but she had tachypnea with a respiratory rate of 30 breaths per minute, oxygen saturation of 60% in room air, and she had edema of the right hemiface. On chest auscultation, there was a notable diffuse reduction of vesicular murmur, cardiac sounds were rhythmic and normal sounding, and there were no murmurs. Her calves were free from clubbing, and both Bancroft’s and the Homans signs were negative. 

**Figure 1:**
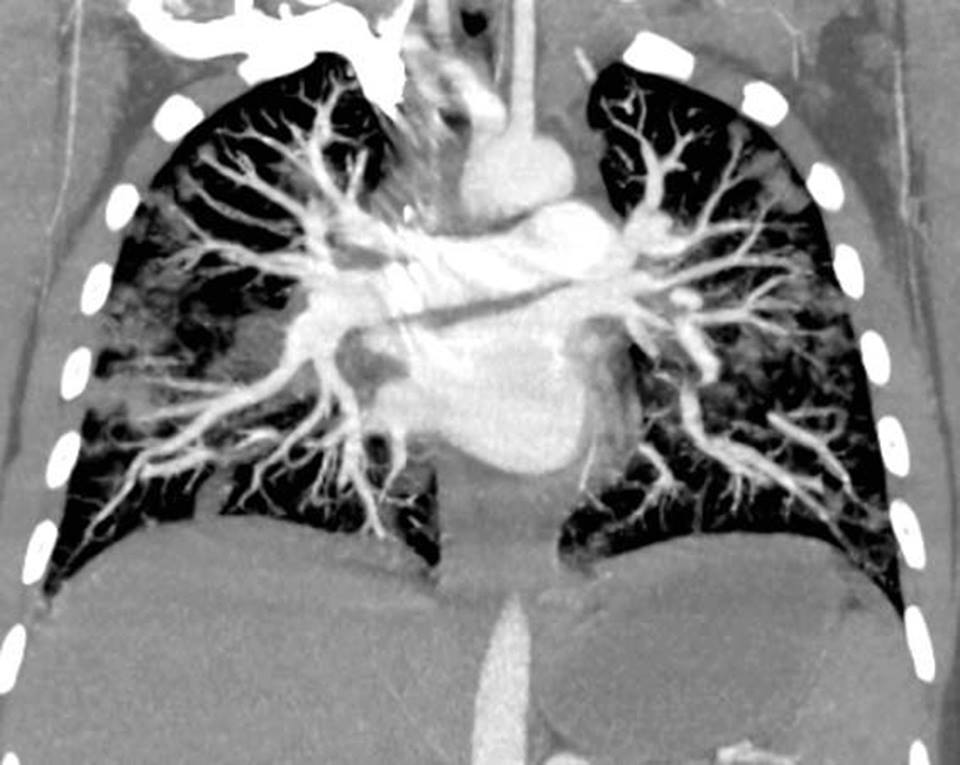
Computed tomography showing pulmonary opacities.

 A hypothesis of pulmonary thromboembolism (PET) was considered and so angiotomography of the thorax was ordered on April 1, 2017 and showed that the patient did not have PET. However, it revealed opaque nodules sparsely distributed throughout the pulmonary parenchyma bilaterally, thickened interlobular septa, with ground glass attenuation, and pleural effusion bilaterally, with a cissural component on the left, suggestive of a diagnosis of septic emboli ( Figure 1 ). Laboratory tests of samples taken on April 2, 2017 revealed Leukocytosis at 16,050, with predominance of segmented cells and no bandemia, while C-reactive protein (CRP) was elevated at 26.3 mg/L. 

**Figure 2:**
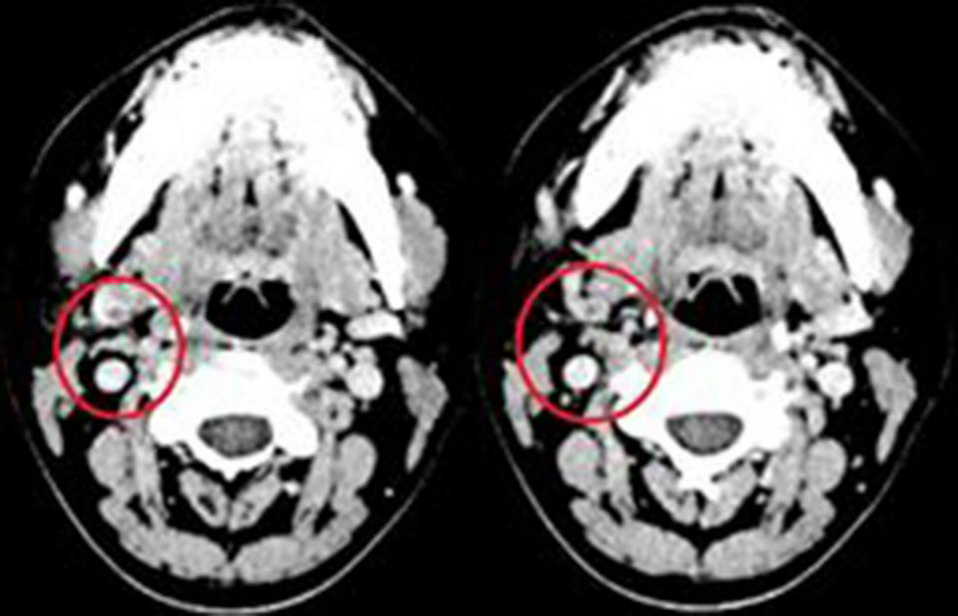
Sequential computed tomography slices showing areas of thrombophlebitis of right internal jugular vein branches (inside red circles).

 On April 4, 2017, computed tomography (CT) of the face and cervical region showed increased density and enlargement of soft tissues in the right hemiface and thrombophlebitis of right internal jugular vein tributaries ( Figure 2 ). Since clinical and radiological findings correlated, a diagnosis of LS was made. 

 Throughout her clinical course, the patient remained in a standard ward and she was medicated throughout her stay with analgesics, non-steroidal anti-inflammatories, and antibiotics. Analgesia was with dipyrone and tramadol, given regularly over the first 5 days. The inflammatory process was managed with 100 mg of ketoprofen every 12 hours for 10 days and 10 mg of prednisone every 12 hours for 10 days. Initial empirical antibiotic therapy was a combination of azithromycin, clindamycin, and ceftriaxone, but once the antibiogram results were in on the second day, this was altered to 600 mg of clindamycin every 6 hours, for 10 days, and 2 g of ceftriaxone once a day for 10 days. 

 Over the course of her hospital stay, the patient’s laboratory parameters improved to the point that, on April 9, 2017, she had 11,330 leukocytes, was free from bandemia, and her CRP had fallen to 1.7 mg/L. Relief from pain was achieved on the first day of admission and after 24 hours the patient no longer exhibited dyspnea and her pulse oximetry reading was 95% in room air. Since she had improved from both clinical and laboratory perspectives, she was discharged from hospital 10 days after admission. 

## DISCUSSION

 In 1936, André Lemierre, described a disease complex that combined anaerobic and septic bacterial infections after tonsillitis. 7 ^-^ 10 Lemierre’s illustration focused on septicemia after angina caused by *Fusobacterium necrophorum*, describing a progression from suppurative peritonsilar infection, through thrombophlebitis of the internal jugular vein, to septic embolization of distant sites, such as the lungs. 11

 The most common site of infection is the palatine tonsils (87.1% of cases). 12 Odontogenic infections, mastoiditis, parotitis, sinusitis, otitis, and infections of the skin or subcutaneous tissues can also be the primary site of infection. 9 ^,^ 12

 Clinical manifestations include fever with temperatures from 39 to 41 °C and shivering from 4 to 5 days after onset of pharyngitis. 6 ^,^ 7 Pain and stiffness of the neck and cervical lymphadenopathy can also occur. Edema and pain at the angle of the mandible or anterior and parallel to the sternocleidomastoid muscle indicate involvement of the parapharyngeal space (26 to 45% of cases). 6 ^,^ 10 Respiratory problems are present in the majority of cases. 6 In atypical presentations, the patient may not have fever and LS may even not be preceded by pharyngitis. 

 There is pulmonary involvement in up to 97% of cases of the syndrome, 6 ^,^ 8 ^,^ 9 caused by hematogenic propagation of bacteria. 12 Pleural pain may be intense and dyspnea may be present, 9 ^,^ 11 while auscultation may detect pleural friction rub. A chest X-ray typically shows bilateral opacities and small pleural effusions. 9

 Early diagnosis is vital to prevent sepsis and death 3 ^,^ 7 ; but it is very often delayed because of the indolent course and because the syndrome is not well-known. 7 Definitive diagnosis can be made with CT, phlebography, simple echography, or duplex scanning of the cervical region. 6 The most useful of these for diagnosis is CT with contrast, 6 ^,^ 7 which will show edema of soft tissues and filling failures or even the thrombus itself in the interior of the internal jugular vein. 

 The first line treatment for LS is intravenous antimicrobial therapy, 4 with coverage for anaerobic microbes. 6 ^,^ 10 ^,^ 13 Response to antibiotics is slow and the average time between start of treatment and resolution of fever varies from 8 to 12 days. 9 ^,^ 10

 Surgical exploration with ligature and excision of thee internal jugular vein is rarely necessary, but may be indicated in cases with persistent septic emboli or for surgical drainage of abscesses or pulmonary empyema. 6 ^,^ 9 ^,^ 11 ^,^ 14 The role of anticoagulation is still controversial and there are no randomized trials that support its use. 4 ^,^ 6 ^,^ 9 ^,^ 11 ^,^ 14

## CONCLUSIONS

 In view of the potential mortality of LS, it is very important that physicians are able to recognize this syndrome early, primarily after presentations suggestive of pulmonary embolism subsequent to bacteremia of the upper airways, so that they can promptly initiate an effective treatment approach. 
