# Supplementary material for: Lemierre syndrome: case report
Source: J Vasc Bras. 2018 Oct-Dec;17(4):337–40. doi: 10.1590/1677-5449.002418 (PMC6375262; doi:10.1590/1677-5449.002418)
Supplement: Translation

# Translation: Síndrome de Lemierre: relato de caso

**Authors:** Rodrigo de Oliveira Veras, Linda Luísa Barasuol, Carolina Pedrassani de Lira, Flávia Caroline Klostermann, Lourenço Sabo Müller, Luiz Eduardo Nercolini, Gustavo Fabiano Nogueira

**Affiliations:** 1 Faculdade Evangélica do Paraná – FEPAR, Curitiba, PR, Brasil.; 2 Hospital Universitário Evangélico de Curitiba – HUEC, Curitiba, PR, Brasil.; 3 Instituto Neurológico de Curitiba – INC, Curitiba, PR, Brasil.

**Keywords:** síndrome de Lemierre, extração dentária, tromboflebite, embolia pulmonar

## Abstract

A síndrome de Lemierre caracteriza-se pela tromboflebite séptica da veia jugular interna, após uma orofaringite, com embolização séptica para o pulmão ou outros órgãos. Neste relato de caso, apresentamos uma paciente feminina, 37 anos de idade, com história de edema e dor em hemiface direita há três dias, associada a fadiga e dispneia progressiva há um dia. História de extração dentária do elemento 48 há três dias. No exame físico admissional, apresentava-se taquipneica, saturando 60% (em ar ambiente), com edema em ângulo da mandíbula direita, redução difusa do murmúrio vesicular e panturrilhas sem empastamento. Angiotomografia de tórax e exames laboratoriais foram compatíveis com quadro de embolia séptica, e tomografia computadorizada da cervical corroborou o diagnóstico de tromboflebite séptica da veia jugular interna. Foi tratada com antibióticos e sintomáticos. A síndrome de Lemierre afeta mais homens jovens e tem embolização para o pulmão em até 97% dos casos. Extrações dentárias raramente podem ser a etiologia dessa síndrome. A tomografia computadorizada é o método de imagem mais utilizado no diagnóstico, e o tratamento é, essencialmente, com antibióticos; portanto, a abordagem cirúrgica é raramente necessária.

## INTRODUÇÃO

 A síndrome de Lemierre (SL) se caracteriza pela tromboflebite séptica da veia jugular interna, após um quadro de orofaringite, com embolização séptica para o pulmão ou outros órgãos 1 ^-^ 3 . Extrações dentárias também podem originar essa síndrome 4 , porém são extremamente raras 5 . 

 Essa síndrome afeta principalmente adultos jovens 6 e pode ser potencialmente fatal 1 ^,^ 2 ^,^ 4 ^,^ 7 . A SL é também chamada de “doença esquecida”, pela sua raridade 7 , e apresenta uma incidência de cerca de 3,6 por milhão de pessoas por ano 2 , com uma mortalidade de cerca de 5%, quando diagnosticada 5 ^,^ 8 . O tratamento se baseia basicamente em antibioticoterapia abrangendo o patógeno envolvido e raramente necessita de abordagem cirúrgica 6 . 

 Neste relato de caso, apresentamos uma mulher que manifestou a SL, atipicamente após uma extração dentária. 

## RELATO DO CASO

 Paciente do sexo feminino, 37 anos de idade, admitida por procura direta no serviço de pronto-atendimento hospitalar, em 01/04/2017, devido a queixa de edema e dor facial em hemiface direita há 3 dias, associado a astenia e dispneia progressiva aos moderados esforços há 1 dia. Negava episódios de febre. Previamente hígida, realizava atividades físicas diárias sem queixas, anteriormente ao quadro. Relatava história de bruxismo, o qual complicou com trauma dentário do segundo molar inferior direito há 3 dias, necessitando extração dentária, sendo esta realizada de imediato. 

 Ao exame físico geral, apresentava-se em regular estado geral, taquipneica com frequência respiratória de 30 incursões por minuto, saturação de oxigênio de 60% em ar ambiente e edema em hemiface direita. Na ausculta torácica, notou-se redução difusa do murmúrio vesicular, bulhas cardíacas rítmicas e normofonéticas, sem sopros. As panturrilhas estavam livres de empastamento, e os sinais de Bancroft e Homans foram ambos negativos. 

 Foi cogitada a hipótese de tromboembolismo pulmonar (TEP) e, por isso, requisitada angiotomografia de tórax em 01/04/17, que não evidenciou TEP. Porém, revelou opacidades nodulares esparsas pelo parênquima pulmonar bilateralmente, septos interlobulares espessados, com áreas de atenuação em vidro fosco, e derrame pleural bilateral, com componente cisural à esquerda, sugerindo o diagnóstico de embolia séptica ( Figura 1 ). Os exames laboratoriais coletados em 02/04/17 evidenciaram leucocitose de 16.050, com predomínio de segmentados, sem bastonetose, e proteína c-reativa (PCR) elevada de 26,3 mg/L. 

**Figura 1:** Tomografia computadorizada mostrando múltiplas opacidades pulmonares.

 No dia 04/04/17, foi realizada tomografia computadorizada (TC) de face e cervical, que demonstraram a presença de densificação e aumento de partes moles em hemiface direita, além de tromboflebite das tributárias da veia jugular interna à direita ( Figura 2 ). Assim, pela correlação clinicorradiológica, foi determinado o diagnóstico da SL. 

**Figura 2:** Cortes sequenciais de tomografia computadorizada evidenciando, no círculo vermelho, áreas de tromboflebite dos ramos da veia jugular interna à direita.

 Durante toda a evolução do quadro, a paciente permaneceu internada em regime de enfermaria e foi medicada, durante todo o período, com analgésicos, anti-inflamatórios não esteroidais e antibióticos. A analgesia foi realizada com dipirona e tramadol, regularmente durante os 5 primeiros dias. Utilizou-se, para o quadro inflamatório, cetoprofeno 100 mg, a cada 12 horas, por 10 dias e prednisona 10 mg, a cada 12 horas, por 10 dias. A antibioticoterapia empírica iniciada foi a associação entre azitromicina, clindamicina e ceftriaxona, porém, com a reorientação pelo antibiograma logo no segundo dia de internamento, manteve-se apenas clindamicina 600 mg, a cada 6 horas, por 10 dias e ceftriaxona 2 g, uma vez ao dia, por 10 dias. 

 Na evolução, durante os dias de internação, houve melhora dos parâmetros laboratoriais, até que no dia 09/04/17, a paciente apresentava-se com 11.330 leucócitos, sem bastonetose, e com PCR de 1,7 mg/L. A melhora das queixas álgicas foi obtida logo no primeiro dia de internação e, após as primeiras 24 horas, a paciente já não apresentava mais dispneia e a oximetria de pulso era de 95% em ar ambiente. Assim, obtendo melhora clínica e laboratorial, foi concedida alta hospitalar após 10 dias de internamento. 

## DISCUSSÃO

 André Lemierre, em 1936, descreveu o complexo da doença que combina infecções bacterianas anaeróbicas e sépticas pós-amigdalite 7 ^-^ 10 . O foco ilustrativo de Lemierre foi a septicemia pós-angina devido ao *Fusobacterium necrophorum*, descrevendo, então, a progressão da infecção supurativa peritonsilar com a tromboflebite da veia jugular interna, seguida de embolização séptica a distância, como para o pulmão 11 . 

 O principal sítio de infecção são as amígdalas palatinas (87,1% dos casos) 12 . Infecções odontogênicas, mastoidites, parotidite, sinusite, otite, pele ou tecido subcutâneo também podem ser o sítio primário de infecção 9 ^,^ 12 . 

 As manifestações clínicas incluem febre de 39 a 41 °C e calafrios depois de 4 a 5 dias do início da faringite 6 ^,^ 7 . Dor e rigidez no pescoço e linfadenopatia cervical também podem ocorrer. Edema e dor no ângulo da mandíbula ou anterior e paralelo ao músculo esternocleidomastoideo refletem o acometimento do espaço parafaríngeo (26 a 45% dos casos) 6 ^,^ 10 . Alterações respiratórias estão presentes na maioria dos casos 6 . A paciente apresentada atipicamente não teve febre, nem a SL precedida por quadro de faringite. 

 O envolvimento pulmonar nesta síndrome acontece em até 97% dos casos 6 ^,^ 8 ^,^ 9 , devido à propagação hematogênica da bactérias 12 . Pode ocorrer dor pleurítica intensa com dispneia 9 ^,^ 11 , e crepitações finas localizadas e atrito pleural podem ser auscultados. A radiografia de tórax mostra tipicamente múltiplas opacidades bilaterais e pequenos derrames pleurais 9 . 

 O diagnóstico precoce é vital para prevenir sepse e morte 3 ^,^ 7 ; porém, é muitas vezes atrasado devido ao curso indolente e ao desconhecimento da síndrome 7 . O diagnóstico definitivo pode ser feito com TC, flebografia, ecografia simples ou *duplex scan* da região cervical 6 . Destes, a TC com contraste é o exame mais útil para diagnóstico 6 ^,^ 7 , demonstrando edema de partes moles e defeitos de enchimento ou o próprio trombo no interior da veia jugular interna. 

 O principal tratamento para SL é a terapia antimicrobiana intravenosa 4 , com cobertura para anaeróbios 6 ^,^ 10 ^,^ 13 . A resposta aos antibióticos é lenta, e o tempo médio entre o início do tratamento e a resolução da febre varia de 8 a 12 dias 9 ^,^ 10 . 

 A exploração cirúrgica com ligadura e excisão da veia jugular interna é raramente necessária, podendo ser indicada nos casos com embolia séptica persistente e também na drenagem cirúrgica de abscessos ou empiemas pulmonares 6 ^,^ 9 ^,^ 11 ^,^ 14 . O papel da anticoagulação ainda é controverso, não existindo trabalhos randomizados que apoiem seu uso 4 ^,^ 6 ^,^ 9 ^,^ 11 ^,^ 14 . 

## CONCLUSÃO

 Devido à potencial mortalidade da SL, é de grande importância que os médicos possam reconhecer essa síndrome precocemente, principalmente após quadros sugestivos de embolia pulmonar subsequentes a bacteremia em via aérea superior, para que consigam uma abordagem terapêutica precoce e eficaz.
